# Artificial intelligence transforming the publishing industry: a case of the book sector in Africa

**DOI:** 10.3389/frma.2025.1504415

**Published:** 2025-05-30

**Authors:** Justin Salani, Mass Masona Tapfuma

**Affiliations:** Department of Publishing Studies, National University of Science and Technology, Bulawayo, Zimbabwe

**Keywords:** educational book publishing, artificial intelligence, book sector, Africa, digital publishing, textbook publishing, disruptive technologies, emerging technologies

## Abstract

The increasing availability and accessibility of artificial intelligence have triggered a seismic transformation of the publishing value chain, presenting unprecedented opportunities and challenges for publishers. Artificial Intelligence (AI) has been integrated into the entire publishing value chain, streamlining the processes of content acquisition by authors and publishers, content and product development, as well as the marketing and distribution of products. However, the disruptive force of AI renders some publishing functions obsolete and transforms the production and distribution of materials, and subsequently, knowledge dissemination. Despite the acknowledged value and potential of integrating especially generative artificial intelligence in the publishing industry, concerns have been raised over integrity, copyright and intellectual property rights in AI-generated content, text and data mining. The impetus of this study emanates from a dearth of literature on the adoption, challenges and opportunities associated with the integration of AI in the production, dissemination and distribution of publications in the book sector in Africa. This paper explores the role of artificial intelligence in the production and distribution of teaching and learning materials by educational publishers. Informed by the disruptive innovation theory, this conceptual paper provides a review of the extant literature on the integration of artificial intelligence in the educational publishing value chain in Africa and its implications on creativity, integrity and intellectual property rights issues associated with AI-generated content. The paper will proffer recommendations.

## Introduction

Advancements in technologies in the recent past have contributed to the exponential transformations in the book publishing sector across the globe, from digital communication systems to the currently most hyped generative artificial intelligence (AI) systems. The term “publishing sector” in this paper refers to “all companies conducting academic, education and consumer publishing activities” (Frontier Economics, 2020, p. 12). Publishers in Sub-Saharan Africa are fast adopting digital technologies for publishing and distributing their books (Isong, 2023); thereby raising hopes of broadening the reach of and access to the storytelling landscape in the region. Publishers such as NENA (Senegal), AkooBooks (Ghana), and OkadaBooks (Nigeria) are publishing online audiobooks which are downloadable on an MP3 player and ebooks to be read on an e-reader, mobile phone or computer. The African publishing sector continues to face formidable challenges affecting the survival and sustainability of businesses; ineffective marketing, promotion and distribution networks, piracy, lack of capital, high manufacturing costs, poor reading culture and unfavorable book policies where they exist. The rapidly changing, unpredictable digital landscape has challenged publishers to rethink their approaches to publishing and integrate emerging technologies, including Artificial Intelligence (Charkin and House, 2023). Artificial intelligence is a computer system that reproduces human cognition using data that is accessed from a variety of different sources or systems to make decisions and learn from the resulting patterns (Hassani et al., 2020, p. 145). In the context of publishing, AI covers machine learning technology, combined with text, image, and vice-related technologies such as natural language processing, voice recognition or computer, and deep learning (Frontier Economics, 2020). Due to the integration of AI, traditional methods of content acquisition, development, distribution, and marketing have undergone a significant transformation, altering the entire value chain (Saikaly, 2023; Gould and Frankfurter, 2019). A value chain is simply a succession of stages in creating a product or service until it reaches the consumer. The publishing value chain is often referred to as the publishing process, that is, sourcing content from authors (content creators), production (editing, proofreading, design, layout, printing, and binding), marketing, and distribution (wholesalers, vendors, and booksellers). Publishers aim to make a profit with each book they decide to add value to and avail it as a perfect product to the consumer, therefore they have to be meticulous in executing the publishing activities.

The seismic transformation of the publishing value chain because of the increasing availability and accessibility of AI systems has streamlined content acquisition by authors and publishers, content and product development, marketing and distribution of products. This presents unprecedented opportunities and challenges for publishers resulting in professionals in the sector remaining skeptical while some are terrified by the technology. The AI tools that have transformed traditional publishing practices in the value chain include Eltwater, ChatGPT, PublishDrive, ProwritingAid, Grammarly, Wiser and so on. Gaigher et al. (2014) professed that AI technologies can transform the existing production and distribution systems; they can be coined as disruptive technologies in the traditional publishing environment (Păvăloaia and Necula, 2023). AI can be used in content creation (creative writing), translation, production, chatbots and user interaction, recommendations, promotion, marketing and sales, and analytics. Integration of AI in publishing is no longer a question of the future, but a present reality; educational publishers in Africa have to brace themselves and invest in AI for their survival and increase efficiency and effectiveness in their operations (Huston, 2022; Zhao and Prabhashini, 2019; Ngaruiya et al., 2023.

The term “disruptive technology” was coined by Christensen (1997), referring to a newly developed technology that substantially modifies existing technologies, the usual way of doing operations and business models (Satalkina and Steiner, 2020; Zighan, 2022). In addition, Marquardt et al. (2017), opine that disruptive technology often destroys or reshapes an existing environment, and replaces obsolete systems with modern, favorable, and more convenient solutions. Isong (2023) reported that despite the numerous creative writing workshops hosted in Sub-Saharan Africa and facilitated by renowned authors like Chimamanda Ngozi Adichie to create “intimate spaces” for budding writers, the output remains low in some spaces. Kenya is reported to experience low production; they have good writers but are not writing. The authors expressed that because they are busy trying to earn a living through other means; they do not have time to write. AI tools enable publishers to streamline their workflows, reduce costs and improve the quality of their content, as they allow them to automate certain tasks performed by humans (Vinay, 2023). As AI continues to disrupt the traditional publishing landscape, publishers' survival hinges on their ability to embrace these technologies and adapt to the rapidly changing environment (Brownrout et al., 2023). Many writers in Africa have resorted to self-publishing to have total control of their works, including the design, content creation and promotion (Isong, 2023). They can leverage the affordances of AI technologies to improve the quality of their products. Disruptive digital technologies are cheaper and simpler for this disadvantaged group including small and upcoming publishers who cannot afford to employ or engage the services of professional editors, proofreaders, designers and so on; AI tools can be adopted to carry out the expensive tasks.

Despite the acknowledged value and potential of integrating generative artificial intelligence in the publishing industry, concerns have been raised over integrity, copyright and intellectual property rights in AI-generated content, text and data mining. The limited availability of literature on the adoption, challenges and opportunities associated with the integration of AI in the production, dissemination and distribution of publications in the publishing sector in Africa prompted the need for this study. The paper explores the role of artificial intelligence in the production and distribution of teaching and learning materials by educational publishers; approximately 95% of books produced in Africa are textbooks mainly due to the large numbers of consumers driven by the book-pupil ratio of 1:2 (one book for every 2 learners). Publishers rely on government purchases of textbooks for schools, so publishing fiction and general books is a risky venture. Most African countries continue to experience economic recession which affects the viability of the publishing business; a situation compounded by the lack of a reading culture among Africans and consumers' lack of disposable income to purchase books for leisure reading. AI can be adopted as a cost-reduction measure, in addition to increased product quality, efficiency, and effectiveness in the publishing value chain activities. Informed by the Innovation Diffusion Theory, this conceptual paper reviews the extant literature on the integration of artificial intelligence in the educational publishing value chain in Africa and its implications on creativity, integrity, and intellectual property rights issues associated with AI-generated content. The paper commences with a discussion on the Innovation diffusion theory and articulates the methodology employed to retrieve literature for the systematic review, followed by the literature review and discussion of the literature, conclusion, and recommendations.

## Theoretical framework

Theories play a fundamental role in explaining how natural or social experiences occur by highlighting the key drivers and outcomes of the intended occurrence (Bhattacherjee, 2012, p. 26. In this study, the innovation diffusion theory (IDT), formulated and further developed by Rogers (1983) is adopted to facilitate understanding of issues around the acceptance and adoption of AI technologies in the educational book publishing sector in Africa.

An innovation is an idea, practice, or object that an individual or unit of adoption regards as new (Rogers, 2003) while diffusion is the process of *communicating* an innovation through selected *channels* over *time* among members of a *social system* (Rogers, 2003). In adopting AI, Stakeholders (publishing value chain) create and share information to have a shared understanding. The four elements highlighted in the definition impact the rate of acceptance and adoption of the innovation. It is important to mention that the diffusion of innovation can be affected by the norms and values of the social system; they can facilitate or inhibit adoption. Diffusion aims to effect change in the function of a social system, therefore, the presence of experts and change agents is critical as they influence the rate of adoption (Bhattacherjee, 2012, pp. 31–32). The IDT suggests that a 5-step process is followed in innovation decision-making, starting with knowledge (awareness) of the innovation and its functions, followed by persuasion; in this stage information about the new technology from trusted sources is shared to minimize uncertainties and convince them to accept or reject the innovation. Knowing the strengths and weaknesses of the innovation is important for stakeholders to be aware of “the changes that occur in an individual or a social system as a result of the adoption or rejection of an innovation” (Rogers, 2003, p. 436). The third stage is making the decision; individuals engage in activities that lead to a resolution to adopt or reject the innovation, e.g., trialing the innovation. The implementation stage follows if the decision to adopt the technology is taken, i.e., the innovation is used. This is done with the assistance of a technical team to deal with issues that may arise with the technology. Lastly, confirmation to adopt or discontinue the innovation is made depending on the attitude of the individuals.

The IDT pinpoints five elements of an innovation that assist in explaining the relative speed of adoption by members of a social system, namely; relative advantage, compatibility, complexity, observability, and trialability. The rate of adoption is measured by the number of individuals who adopt the technology within a given time. *Relative advantage* refers to the level to which an innovation is regarded as better than the idea it surpasses; stakeholders consider the benefits they will derive from it. Economic and social factors are measured including convenience and satisfaction. *Compatibility* refers to the level to which an innovation is believed to be consistent with the existing values, past experiences, and needs of prospective adopters (Rogers, 2003). If the innovation is not compatible with the norms and values of a social system, its adoption is inevitably slow. *Complexity* refers to the level to which an innovation is viewed to be difficult to understand and use. When an innovation is easy to use, individuals adopt it much faster than a complicated one which would require adopters to acquire new skills and understanding. *Trialability* refers to the level to which an innovation can be tested to remove uncertainties in the individuals. *Observability* refers to the extent to which the effects of the innovation are visible to others and the results are tangible. The easier it is, the higher the chances of adoption.

## Methodology

**Figure 1:**
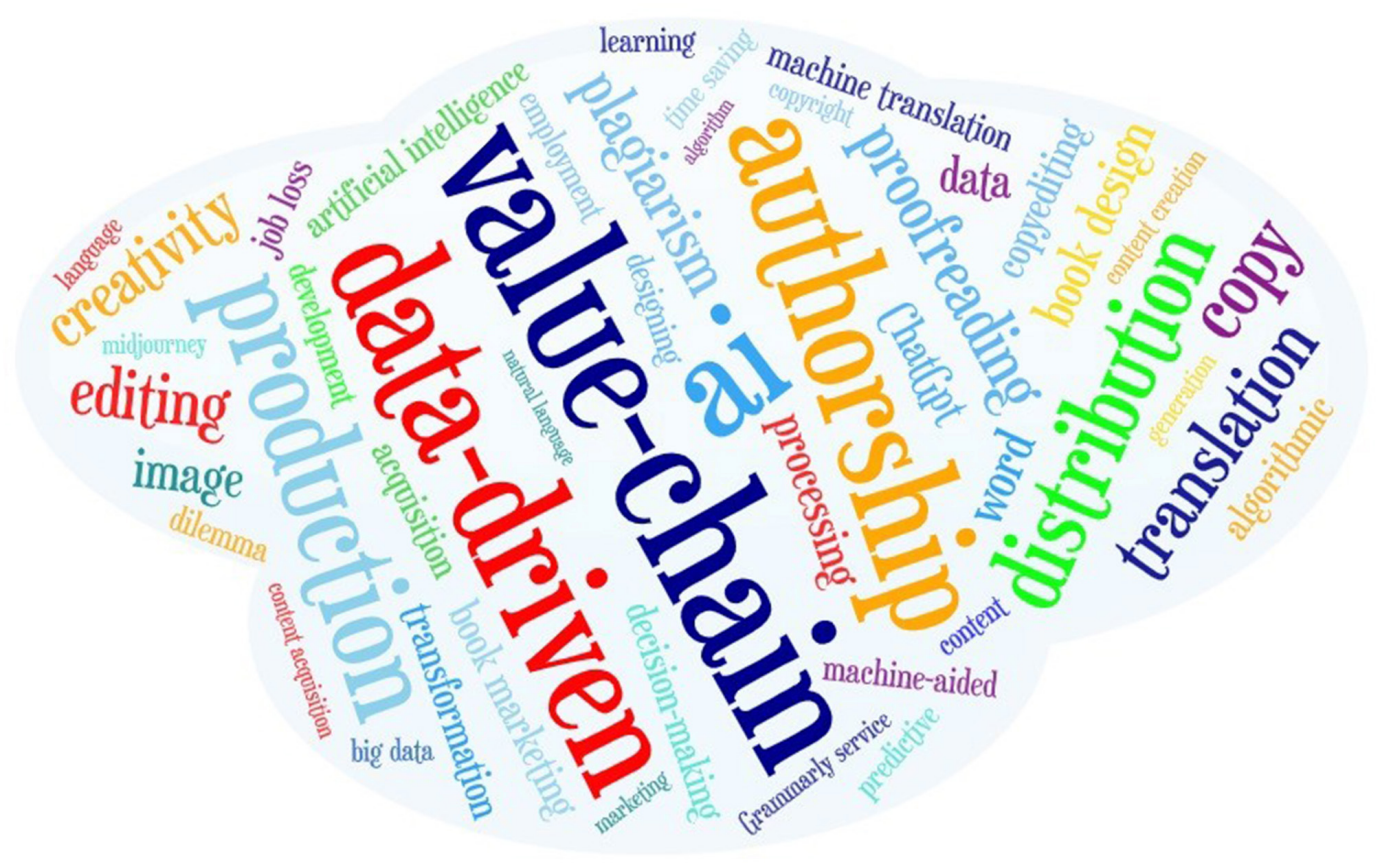
Codes and themes generated.

This study used a qualitative approach to gain insight into how Artificial intelligence can be incorporated into the book publishing value chain and disrupt the educational book publishing sector. Secondary data were collected via a review of existing literature. A secondary data analysis technique was employed to achieve this goal, using a literature review. Secondary data sources were identified and collected through a comprehensive literature search for peer-reviewed scholarly articles. The study was limited to full-text articles which were accessible and focused primarily on AI and publishing in Africa. The review was limited to articles published in English between 2019 and 2024 which were retrieved using the following search terms: educational book publishing, Artificial intelligence, Book publishing industry, digital technologies, Textbook publishing and Book sector. Articles that were considered as relevant focused on educational publishing in Africa, the application of artificial intelligence in book publishing in Africa, and the transformative impact of artificial intelligence on the book value chain. Articles that focused on the various aspects of the book value chain such as manuscript acquisition or commissioning, editing or proofreading, designing or production, marketing and distribution were included in the analysis. The databases used for the literature search included Elsevier Science, Google Scholar, Science Direct, Research Gates, Web of Science, and Springer Link. A total of 60 articles were retrieved comprising 53 journal articles, three white papers, two survey reports, and two theses. All the retrieved documents were relevant to the digital publishing and usage of AI in the book publishing value chain. Only 12 sources (see Table 1) were related to the African publishing industry. Although theses, white papers and reports were relevant and informed the study, they were not included in the analysis; only journal articles were considered as they enabled the researchers to make informed inferences. To avoid bias, the researchers ensured preliminary peer reading to ensure that the articles were relevant to the study and that a balance of arguments could be achieved. The researchers also identified articles from different geographical regions in Africa. Objectivity was maintained throughout the analysis, ensuring that diverse scholarly perspectives, from positive to negative were captured. A content analysis of the literature was done to identify common themes and patterns in the literature related to the adoption and usage of AI in the publishing value chain including challenges and opportunities it presents to Educational book publishing. Secondary data gathered from the retrieved articles were deductively and manually coded and thematically analyzed. Themes identified include: AI simplifies data analysis, AI improves algorithmic decision making, AI streamlines content acquisition, AI improves writing and editing, AI enhances productivity, AI enhances machine translation, integration of machine-aided book design, use of AI tools for copyediting, AI use in book marketing, AI leading to job displacement, AI creating copyright dilemmas, AI stifling creativity and AI resulting in loss of integrity (see Figure 1). This study is limited by the availability and quality of secondary data sources while the findings are based on the existing literature in the field and may not reflect all recent developments in the book sector in Africa.

**Table 1:** Findings.

**Author(s)**	**Purpose of the study**	**Study design/methodology used**	**Key findings**
Adaka and Olubiyi, 2022	The paper examines the challenge AI poses to authorship and inventorship under copyright and patent laws	Doctrinal method of legal research	Under the extant legal framework in Nigeria, AI cannot be designated as an author or inventor AI-created works are excluded from intellectual property protection in Nigeria
Afolabi and Zolkepli, 2023	The study explores the adoption of big data in the Nigerian book publishing industry	Literature review	The study reveals the use of big data for publishing development in developed countries There was no evidence of big data adoption in the Nigerian publishing sector Big data can help publishers increase the effectiveness of their book publishing processes
Afolabi and Jimoh, 2024	The study investigates the influence of AI on the Nigerian publishing business, with ChatGPT as the reference point	Literature review	AI can improve the publishing process for books as it provides tools for improved writing, editing, production, distribution, and marketing AI presents difficulties, including the requirement for publishers to adopt new technologies and the possible threat to the careers of publishing professionals
Al Sawi and Alaa, 2024	The research aims to investigate editors' and proofreaders' perception of existing AI tools for editing and proofreading The paper examines whether editors or proofreaders view AI as an opportunity or a threat and considers insights into the future of AI tools for them	Qualitative method	Editors and proofreaders are using AI tools such as Grammarly, Perfectit, QuilBot, Trinka, Hemingway Editor and ChatGPT ChatGPT and Grammarly are the most used AI tools AI results in increased efficiency, time-saving, and improved productivity Concerns include that AI can replace humans There are ethical challenges associated with AI There is a need for continued human involvement in the editing and proofreading processes
Bedu, 2024	The study interrogates natural language data drawn from African languages to present current and future challenges, opportunities, and potential for developing AI algorithms that could fit neatly into the translation of African languages	Quantitative and qualitative	Challenges for AI translation of African languages include the fact that the languages do not always have one corresponding semantic object The study also reveals that syntactic operations in African languages do not always have one corresponding semantic operation
Ekhator, 2023	The paper examines the extant legal framework on copyright law and juxtaposes it with the current advancements in artificial intelligence system	Doctrinal research method	Based on the current position of the copyright law, AI are not legal persons and cannot own the intellectual property right in works generated by them
Heintz et al., 2022	The paper compares the accuracy and effectiveness of Word Service AI Proofreader to expert proofreading by human editors and two other popular proofreading applications—automated writing analysis tools of Google Docs, and Microsoft Word.	Comparative analysis	Wordvice AI proofreader achieved performance levels at or near that of the human editors, identifying similar errors and offering comparable suggestions in the majority of sample passages. The Wordvice AI proofreader also had higher performance and greater consistency than that of the other two proofreading applications evaluated.
Myagila and Kilavo, 2021	The study compares the performance of SVM and CNN in translating sign language through image recognition	Principal content analysis	CNN has a higher performance score (96%) in all of the parameters which are accuracy, recall and precision while SVM scored a similar rate in precision but lagged in precision and accuracy
Ngaruiya et al., 2023	The study explores the adoption and use of artificial intelligence in Kenya's digital creative sectors. The paper also documents ways in which AI is transforming traditional workflows, job roles, and skill requirements	Qualitative, semi-structured interviews	There is a rapid adoption of AI by Kenyan creators Ghostwriters, graphic designers and coders use AI to perform various functions within their portfolio There are, however, concerns about job security, ethical implications and the need for upskilling
Onoja, 2023	The study aimed to explore the impact of AI on the Nigerian writing industry, with a focus on the opportunities and challenges that arise as a result	Mixed methods approach	AI has the potential to transform the writing process by providing writers with tools for improved writing, editing, and publishing However, AI integration threatens jobs, and there is a need for writers to adapt to new technologies
Emezue and Dossou, 2021	The study focused on the task of multilingual machine translation for African languages	Multilingual machine translation model	The study introduced a novel back translation and reconstruction objective to leverage monolingual data effectively
Khan and Gotora, 2023	The article provides insight into the existing jurisprudence surrounding AI and copyright in the South African context	Qualitative	South Africa might lack the legal framework to tackle the future implications of conferring authorship on non-human entities

## Literature review and discussion

The hype surrounding AI and its implications on various economic sectors, including the publishing industry, was catalyzed by Open AI's launch of Generative Pre-trained Transformer-3 (GPT-3) in 2020 (Kulesz, 2023). Subsequently, Large Language Models (LLM) became a critical influence in the publishing of academic books, journals and related materials (De Angelis et al., 2023). The publishing industry is on the verge of a seismic shift, driven by generative AI's ability to churn out unique content with computer speed (Brownrout et al., 2023). Concurrently, Bhaskar (2020) asserts that AI's impact on publishing is far-reaching, affecting both business operations and content creation. The author further articulated that advanced AI like Google's Gemini and Open AI's ChatGPT can now translate and even write text, blurring the lines between human and machine authorship, hence a need for publishers to adapt to this new reality where machines contribute to the core product. According to Vinay (2023), the adoption of AI tools has enabled publishers to streamline their workflows, reduce costs and improve the quality of their content, as these tools allow them to automate certain tasks done by humans. The integration of AI in publishing therefore is no longer a question of the future, but a present reality, and educational publishers in Africa have to continue investing in AI for their survival, given the disruptive nature of such emerging technologies (Huston, 2022; Zhao and Prabhashini, 2019; Ngaruiya et al., 2023). This section will discuss the findings of the extant literature on the role of AI in educational publishing in the global sphere narrowing down to Africa. The development of technologies in publishing; and AI integration in the various processes of the publishing value chain, including content acquisition, development, authorship and translation, production and marketing are discussed in this section. AI challenges for the book sector in Africa, as well as the conclusions and recommendations, are presented in this section of the paper.

## Integration of technologies in the publishing business

Technology integration in the production and distribution of electronic materials can be traced from Project Gutenberg in the 1970s and Michael Hart is commonly credited as the undisputed father of the e-text (Ballard, 2012; Bashorun et al., 2013). Computers were first used to assist printing of abstracting and indexing services (Bashorun et al., 2013). The first digital file, therefore, was the United States Declaration of Independence, which was a product of Project Gutenberg (Gaigher et al., 2014). The developments in computer technologies, including e-readers, the World Wide Web (WWW) and the arrival of the Portable Document Format (PDF) in 1993 further revolutionized the production and distribution of reading materials (Acosta-Vargas et al., 2017). According to the [World Intellectual Property Organization (WIPO) (2021)], the technological aspects of the digital revolution have affected the way books and other publications are produced, priced, promoted, and distributed, with notable changes in the value chain. In addition, the seismic shift from print and paper to digital ink and screen has resulted in new business models that challenge traditional hierarchies, allowing authors to self-publish and readers to become content creators, thus blurring the lines between roles, leading to a new ecosystem where the traditional publisher is no longer the sole gatekeeper (Murray and Squires, 2014).

The digital distribution of published materials, influenced by datafication, and algorithmic selection, and the platformisation of literary works have influenced publishing (Spjeldnæs, 2022; Nielsen and Sarah, 2022). In addition, the advent and proliferation of social media platforms have reshaped book production, distribution and marketing, with new players becoming a part of the value chain (Krumova, 2017). It is apparent that social media, data analytics, algorithmic filtering and AI-based precision decision-making have become essential aspects in the development and distribution of publications, but have also exacerbated the divide between small and large publishers (Nolan and Dane, 2018). Chen (2023) contends that the publishing industry has undergone significant changes since the first digital replica in the form of e-books appeared online in 1996, as social media platforms have gained momentum, impacting book development and distribution. In addition to other new media platforms that continue to shape the book publishing industry, blogs have become a 21st-century publishing phenomenon, influencing especially the distribution component of the publishing process (Elega and Ozad, 2018). Speaking at the Business of Books Conference held on 31 October 2024 in Harare, Emmanuel Choga, of Akello, urged African publishers to work together to embrace the digital age, position themselves in the international marketplace on equal terms and magnify the African voice (Ngalame, 2024). This is against the backdrop of a fragmented African publishing industry where authors and publishers face economic constraints and difficulty reaching global and wider audiences to market and sell their books. Therefore, Africa should bridge the divide by harnessing digital distribution channels to increase the visibility and reach of publications from Africa.

Data analytics and algorithmic decision-making, machine learning and AI are increasingly becoming part of the digital transformation agenda in the publishing industry (Saikaly, 2023). Publishers in China, for instance, have quickly followed up on new technological developments such as artificial intelligence, cloud computing and big data, and this presents the overall characteristics of steady, pragmatic and good development (Yang, 2021). Yang adds that the integration of these technologies has resulted in new publishing models and constantly emerging business forms providing rich and colorful reading experiences for readers. The United Kingdom (UK) Frontier Economics (2020) conducted a study to establish the attitudes and behaviors of publishers toward investment in AI in their organizations using stakeholder interviews, three case studies and an online survey sent to members of the association countrywide. The study found that AI is being applied throughout the publishing value chain, with AI tools being used to acquire and develop new content as well as in marketing. As a result of the integration of new technologies, the traditional value chain, where a single publisher handles everything from manuscript acquisition to marketing and distribution is being disrupted and disintermediated at every stage (Murray and Squires, 2014). AI investment decisions were largely influenced by the benefits accrued to both the publishers and consumers from using the technology and that it can be applied in various ways throughout the publishing value chain. Benefits identified include improvement of quality, time-saving, speed and improvement of operational efficiency, cost savings and provision of additional insights for innovation activities, and improvement of the customer experience (Frontier Economics, 2020). In a speech at the Ghana International Book Fair in August 2024, Lawrence Njagi, the chairman of the African Publishers Network (APNET) and also an educational publisher, encouraged publishers to cautiously integrate AI into African systems to promote development and avoid throttling creativity and innovation in future generations (Williams, 2024). The African book sector landscape is a slow adopter of the new and emerging digital and disruptive technologies despite the outlined benefits they would accrue given the formidable challenges faced by the industry, thus hindering their success. The African publishing industry thrives on publishing for the education sector which demands an efficient and timely supply of quality textbooks to schools. Therefore, it is expedient that they embrace AI technologies and put them to good use.

According to Yi (2020), the future of the publishing industry is closely related to the application of big data technology, affecting the operation of various components of the publishing industry's supply chains. Afolabi and Zolkepli (2023) explored big data analytics for publishing development in Nigeria and discovered that big data analytics has been integrated into the book development processes, and processing tools with advanced academic text creation capabilities will continue to impact how authors create content. It is apparent that AI fits into the current and future publishing models (Rutkowska, 2020). In addition, developments in sophisticated technologies have seen the integration of augmented reality and virtual reality by publishers in an attempt to enhance their publishing deliverables; this has been catalyzed by a rise in the popularity of smartphones (Gudinavičius and Markelevičiute, 2020; Elaish et al., 2024). According to Charkin and House (2023), blockchain technology, generative AI and augmented reality have had an impact on the publishing industry in its entirety. This shows that publishers are making significant strides to keep pace with technological changes. The absence of literature on strides made by African publishers in using big data technologies besides those in Nigeria is enough to encourage the value chain stakeholders to embrace it for the good of the companies and their consumers by increasing their visibility and reach.

## Educational publishing and integration of AI in the textbook value chain

Educational publishing involves the creation, planning and distribution of textbooks, workbooks, tests and teacher support materials that are used in preschools, primary schools, and secondary schools, both private and public [World Intellectual Property Organization (WIPO), 2021]. It involves the origination, development and distribution of educational materials for primary and secondary schools, often developed following a specific national curriculum or international standards [Bläsi, 2018; World Intellectual Property Organization (WIPO), 2021]. As mentioned earlier, publishing businesses in Africa thrive on textbook production and provision to the education sector; that is where large numbers of consumers are and are mostly funded by the government, international non-governmental organizations (NGOs), donors, and parents. Quality education is the key to national development and achievement of the Sustainable Development Goals (SDGs). Textbooks and other learning materials play a significant role in instruction as they are developed following the requirements of the curriculum. Therefore, 95% of publishing output in Africa is in educational publishing and there is very little gain in publishing general and literary books.

The traditional educational book value chain has undergone considerable changes due to the adoption and usage of new technologies, including artificial intelligence (Ho et al., 2011). The traditional publishing value chain was a simple linear process with authors as originators, publishers as moderators and producers, and booksellers as final distributors of the published materials. Traditionally, the publishing process of materials written for the curriculum started with the basic publisher deciding what to publish and what not to publish, culminating in commissioning authors for content development [World Intellectual Property Organization (WIPO), 2021]. The commissioning editors identified and commissioned authors who then developed the manuscript. The publisher was involved in editing, using paper-based or basic on-screen markup systems, and engaged illustrators and designers for book design using basic desktop publishing software, with the production editor overseeing the development of the manuscript (Tian and Martin, 2013). Printing was often outsourced, and the publisher managed multiple channels through which the final book was distributed, including booksellers, agents or government agencies, in the traditional publishing process (Lueke et al., 2013).

In the early 2000s, a radical transformation of the publishing industry, enhanced by the increasing accessibility and affordability of digital technologies transformed the textbook value chain (Øiestad and Bugge, 2014); computational systems, natural language processing tools and neural networks, have rendered some processes in the traditional publishing value chain obsolete (Gaigher et al., 2014; De Angelis et al., 2023). This is confirmed by the findings of a survey by the Frontier Economics (2020), to determine the role of AI in the publishing industry. The study established that AI has permeated all the functions performed throughout the value chain such as content acquisition or commissioning, content and product development, marketing, promotion and sales. Natural language processing tools, machine learning, deep learning and speech recognition have been integrated into the production and distribution of teaching and learning materials. Gould and Frankfurter (2019) perceived that AI has eased several publishing tasks, including; the conversion of paper-based publications to digital content through optical character recognition, automated administration tasks such as handling invoices, payments as well as provided customer services through Chatbots.

Studies have shown that AI has been adopted into the publishing sector in Africa, particularly in West Africa. Afolabi and Jimoh (2024) investigated the impact of generative AI on the Nigerian book publishing industry, specifically ChatGPT. They found that AI can improve publishing functions such as writing, editing, production, distribution and marketing, although it can present challenges for professionals in the book sector. The scholars underscored the need for publishers in Nigeria to adopt new technologies such as artificial intelligence. This is important as AI's disruptive force requires publishers in Africa to take advantage of the technology to cope with daunting challenges facing the industry. As publishers and authors adopt AI in the production of educational materials, there is a need for publishers to upgrade their systems to accommodate both the benefits and challenges associated with the new technology.

A study conducted by Oparinde et al. (2024) to establish the perspectives of members of the National Scholarly Editors' Forum in South Africa on current developments in scholarly publishing, recommended that leading publishers, taking the roles of innovators and early adopters must continuously reassess their publishing processes to accommodate both the benefits and drawbacks associated with the adoption of AI in the industry. This has necessitated the adoption of AI tools to streamline copyright management systems and address the possible infringements that emerge from AI use.

### Content acquisition

In traditional publishing, the editor is vested with the duty to decide what to publish and what not to publish. However, educational publishers' decisions are influenced by the demands of the curriculum. A decision has to be made whether to revise an existing textbook and come up with an updated edition or produce a new title or edition altogether. The decision-making is informed by data; so artificial intelligence comes in handy as it simplifies and speeds up the processes of data analytics for precision algorithmic decision-making (Afolabi and Zolkepli, 2023). Available evidence suggests that AI tools have been used to streamline publishing operations including content acquisition, although the usage of the technology for this function in Africa is still in its nascent stages. This is in contrast to the developments in the Global North where AI tools have been adopted and integrated into all stages of the book production processes, and algorithms have redefined the way publishers, authors and journalists approach the task of generating written material (Saikaly, 2023; Kraus, 2019; Ryzhko et al., 2024; Huston, 2022). AI has also been used considerably in China, to perform publishing functions such as topic or thematic area selection, thus, content acquisition decision-making processes have become machine-aided (Zhao and Prabhashini, 2019). On the contrary, the use of AI for content acquisition and decision-making by African publishers is still limited. Using the Innovations Diffusion Theory, the adoption of AI is due to a combination of factors such as compatibility and complexity (Rogers, 1995). As AI requires large volumes of training data using sophisticated computational models, its adoption in the publishing industry becomes a challenge given that most publishers in Africa operate on constrained budgets (Molapo, 2020), and lack the human capital needed to develop and implement AI systems.

### Content development, authorship and translation

According to Onoja (2023), AI has the potential to enhance the writing process by providing writers with tools for improved writing, editing, and publishing. It is apparent from the findings in Onoja's study that AI is being used in the book sector in Africa, in aiding the writing process, although its use is more evident in East and West Africa. Ngaruiya et al. (2023), in a study of the domestication of AI in Kenya, established that authors have embraced AI-based writing assistant tools for generating content, improving productivity, and meeting client demands. The value of AI for authors lies in its ability to help streamline research, significantly reducing the time spent capturing underlying data and resources to inform the writing process (Frontier Economics, 2020). Onoja (2023) conducted a study to explore the impact of AI on the Nigerian writing industry, with a focus on the opportunities and challenges that arise as a result. The study found that while AI tools are being used in writing, most of the writers involved in the study had not adopted them. Saikaly (2023) notes that traditionally, research and writing involved an investment of time and effort by human authors, but the AI-powered natural language processing algorithms have accelerated the processes as they can generate thousands of words of content in a few seconds. This has transformed publishing practice by allowing publishers to deliver information to their audiences faster and more efficiently. While it is evident that the diffusion of AI in the writing processes in Africa is still in the early stages, it is also important to acknowledge that even globally, most publishers using AI are large multinational publishers with large-scale operations and adequate resources for effective deployment of AI. These include Pearson, McGaw-Hill Education, and Penguin Random House (Frontier Economics, 2020; Saikaly, 2023).

Studies have also shown that AI is being utilized to perform text and sign language translations, thus addressing inequalities in the book sector. Myagila and Kilavo (2021) explored the use of image recognition options for translation purposes in Tanzanian Sign Language; they found that machine learning models have an acceptable rate of accuracy, recall, and precision. Artificial intelligence and machine learning techniques have presented new models for multilingual translations, a system that has the potential to help optimize translation tasks. Emezue and Dossou (2021) introduced a multilingual machine translation system for six African languages. If integrated, the technology can help publishers improve the production and distribution of learning materials for even marginalized languages in Africa. The Adoption of natural language processing tools for translations in Africa has the potential to transform not only communication but also education, where these tools are also used to facilitate translation of educational materials (Bedu, 2024). While concerns have been raised about the job displacements in translations, Jiang and Lu (2021) highlighted the need for complementarity between machine and human translators, hence, human involvement remains fundamental in translation works. Overall, this review has shown that human-machine co-creation of materials is necessary, and machines will not entirely replace humans in content development, authorship, and translations.

### Production

Literature shows that production processes in textbook publishing in Africa have metamorphosed, as emerging technologies, including AI, have been adopted, and machine-aided design and editing are now a reality. Purely AI tools and AI-powered platforms, such as ChatGPT, Grammarly, Quillbot, Jenni and Copy.ai have been adopted by editors to streamline the editorial processes in book production in Africa. Al Sawi and Alaa (2024) conducted a study in Egypt aimed at investigating editors' and proofreaders' perceptions of existing AI tools that can be used in the editorial and proofreading processes. The qualitative study also sought to examine whether editors or proofreaders view AI as an opportunity or a threat and consider their insights into the future of AI tools for them. The study established that editors and proofreaders use AI tools in editing and proofreading, with Grammarly being the most used tool followed by ChatGPT and Perfect. The study also found the benefits associated with the usage of AI tools in editing, to include, time-saving, enhanced quality and productivity compared to paper-based and basic onscreen editing. In addition, tools such as GPTZero and AI Text Classifier were found to be useful for AI plagiarism checks, as they are capable of distinguishing between a text written by AI and another written by a human being (Al Sawi and Alaa, 2024; Wright et al., 2024). Heintz et al. (2022) performed a comparative study of proofreading completed by an AI tool, i.e., Wordvice AI Proofreader by experienced human academic editors. The study found that in most texts analyzed, Wordvice AI Proofreader achieved performance levels at or near that of the human editors, identifying similar errors and offering comparable suggestions in most of the sample passages. However, there is a wide variation in the adoption of AI for editorial processes between African publishers and those in Europe and Asia (Saikaly, 2023). AI is also used to perform translations and adaptation functions in publishing. However, the collaboration of human and neural machine translation is important, especially during the post-editing phase of machine translations (Mohammed et al., 2023). While AI translation of educational materials in Africa is crucial in fostering equitable access to education, challenges exist given the multiplicity and lexical complexity of indigenous languages. This explains why the adoption of machine translation tools in Africa has been low.

It is also apparent from the literature available that designers have adopted AI tools for image recognition and enhanced visual effects (Ngaruiya et al., 2023), but there is very little evidence of AI use for design purposes in Africa even though the technology is being used by publishers in developed countries where access to AI tools is widespread. Book designers use AI for several designs, including layout. It is used to select appropriate color schemes, fonts, and composition, and this automated layout function when optimized with AI can reduce the time and cost to improve efficiency (Sofia et al., 2024). A study conducted by Kukol (2024) involving Ukrainian publishers shows that several publishing houses are already creating book covers with the help of AI tools. Kukol added that publishers are leveraging AI's ability to create unique and attractive book covers using algorithms to analyze key themes and moods of a book. Similar uses of AI in modern book design were also observed among publishers in China (Yu and Cao, 2023). AI tools especially ChatGPT, Midjourney, and Dall-E are being used to generate illustrations even for children's books, enhancing creativity and efficiency; offering an innovative approach that benefits authors, illustrators, and publishers (Hariffadzillah et al., 2023). While there is a fear that AI can replace human illustrators leading to job losses, it is also apparent that human intervention remains necessary, thus confirming the notion that disruptive technologies do not seek to replace existing practices but rather complement them. Midjourney is among the popular AI tools used for generating images and illustrations for books, however, the tool is not available for free, and it requires the use of other design tools such as Photoshop and Illustrator to edit, refine, or incorporate the general images into the final book (Esquivel, 2024). Although AI is still an emerging disruptive technology for African publishers, there is a possibility that the technology will spread from early adopters to maximum utilization for enhanced productivity, depending on factors such as compatibility, cost, and relative advantage.

### Marketing

Natural language processing tools, deep learning and machine learning are among the tools that aid the marketing of published materials, and these tools are being used especially in identifying market trends for demand forecasting to inform publishers' marketing strategies (Frontier Economics, 2020). According to Saikaly (2023), AI has transformed marketing practices, as personalized content recommendations, powered by AI have become crucial for boosting reader engagement and providing algorithmic recommendations tailored to the user's preferences. Saikaly added that traditional market research involved manual data collection and analysis, often a time-consuming task, but AI has emerged as a game changer, enabling publishers to gather and precisely interpret large volumes of data to make data-driven decisions that shape their commissioning or acquisitions, content strategies and distribution channels. It is possible to determine bestsellers, predict user satisfaction through machine learning techniques (Lee et al., 2021), and predict book popularity before publication (Sachdeva et al., 2024). In addition, AI algorithms can be used for book recommendation in online environments to promote book visibility, thereby optimizing the book marketing function (Tegetmeier et al., 2023). However, as in other publishing functions, AI and machine learning applications in book marketing in Africa are still a far reality, as this review has shown that usage of these technologies in Africa is still in the embryonic stages. This is partly because the publishing industry in Africa is largely educational, and decisions are influenced by enrolment figures in schools and less often data-driven and machine-aided. There is however a need for publishers in Africa to take advantage of these technologies as new players are emerging to take part in the lucrative book publishing industry, capitalizing on emerging technologies.

## Implications of AI on creativity, integrity and intellectual property rights

Despite the acknowledged value of AI for the book sector in Africa, its adoption has brought with it challenges for authors, publishers and other relevant stakeholders.

### Creativity and integrity

The integration of AI tools in authorship in particular has ethical implications which require due diligence in the usage of the tools (Ngaruiya et al., 2023). In the same context, Onoja (2023) argues that one of the major concerns surrounding AI-generated content is the potential for plagiarism, and whether AI-produced work should be held to the same standards as human-written content. These issues require careful consideration as AI continues to shape authorship and the entire publishing industry. In their study, Al Sawi and Alaa (2024) found that the use of AI tools raises concerns related to incorrect responses, a potential overreliance on the tools leading to a decrease in the skills of editors and proofreaders, ethical concerns about plagiarism, and the high cost of these tools. Traditionally, authorship belongs to humans who create original works, however, AI algorithms independently generate content, blurring the lines of authorship (Saikaly, 2023). It therefore becomes a challenge for publishers to determine authorship attribution. These challenges which are inherent in the African publishing context require effective policies that support the ethical use of these tools in publishing.

In the trade publishing sector, authors, in conjunction with other creators are likely to face competition from quality, low-cost AI-generated literary materials particularly as self-publishing continues to grow, taking advantage of generative AI's text and image creation capacity (Senftleben, 2023). The author therefore calls for levies that can generate revenue to compensate authors and creators for the loss of revenue, thus preserving the societal value of human creativity. The impact of AI tools on the nature of employment in publishing houses has also been articulated by (Liu, 2023) who argued that AI use can have an impact on the publisher's employment structure, as some job functions may be replaced by machines. Liu highlighted the need for publishing companies and employees to adapt by embracing AI, acquiring new skills, and transforming their careers to remain competitive in this evolving landscape. However, following a survey by Gould and Frankfurter comprising 300 participants including interviews and conversations with professionals in the publishing industry to clarify the concept of AI and its application in business, the researchers argue that investing in AI can result in job stability, as new revenue streams for publishers emerge, hence publishing professionals should be able to adjust and facilitate the effective deployment of AI tools in the organization (Gould and Frankfurter, 2019).

## Intellectual property rights concerns

Adaka and Olubiyi (2022) explored the intellectual property challenges for AI-created works in Nigeria and established that at law, AI cannot be designated as an author, creating a dilemma for authors and publishers about the use of AI-generated text. The unavailability of a specific legal framework excludes AI-created works from intellectual property protection (Adaka and Olubiyi, 2022). Similar challenges, skewed toward authorship were discovered in a South African book sector study by Khan and Gotora (2023) who articulated that South Africa might lack an adequate legal framework to address the future implications of conferring authorship to non-human entities. Ekhator (2023) examined copyright issues associated with autonomous AI machines and found that AI systems are not legal persons, and cannot own their intellectual property rights in works they generate. It also emerged from the study that AI can infringe on the copyright of existing works, where AI generates content similar to existing copyrighted material. This creates challenges for publishers where text generated by AI is used in any publication. While there is a void in the African legislative framework to govern AI-generated works, in countries such as the UK, Hongkong, Ireland, New Zealand, and India, significant steps have been taken to address copyright issues related to AI-generated works as they have amended their statutes to accommodate the copyrightability of works that are machine-generated with human intervention (Gaffar and Albarashdi, 2024). Thus, African publishers should push for the development of such legislative systems in their respective countries to promote the legitimate use of AI tools in the development of teaching and learning materials.

## Other concerns

As artificial intelligence permeates the publishing industry, fears are that AI-driven digital transformation is likely to disrupt the sector causing job losses and transform the role of various stakeholders in the publishing ecosystem. It is likely to bring in new players and displace those that fail to disrupt themselves from within, adjust and adapt. This calls for publishers in Africa to innovate and adjust their business models and create new revenue streams that are anchored by AI. Such slow, but notable developments have been observed in Kenya where Longhorn Publishers has developed and implemented an AI tool to improve student access to learning materials at low cost. The publisher has taken the role of an innovator who will in turn influence others to develop technological solutions that enable them to remain relevant. As AI has demonstrated the characteristics of a disruptive innovation, its adoption and integration in the book sector in Africa and beyond will eventually render some publishing functions obsolete, and at the same time create new functions and new job roles (Ryzhko et al., 2024) that were traditionally not applicable in the industry.

It is evident from the findings that professionals in the publishing industry, such as authors, designers, and marketers are facing an uncertain future as a result of the proliferation of AI tools that can perform the same functions they do. A study conducted in Nigeria by Afolabi and Jimoh (2024), found that AI is a threat for publishing professionals. The study highlighted a need for the transformation of publishing education and training to accommodate the existing changes in technology It is therefore apparent from the findings in this study that the adoption and use of AI in publishing and the book sector in Africa is marred by several challenges, most of which are beyond the influence of publishers. While there are notable differences in AI adoption across the continent, the challenges related to copyright and the fear of job losses are common (Afolabi and Jimoh, 2024; Khan and Gotora, 2023). The legislative framework on copyright and intellectual property in many countries has not been updated to accommodate entirely machine-created works and human-machine co-created works. The fears of AI on jobs have been allayed by arguments that these AI tools cannot work accurately without human intervention. Despite the acknowledged infrastructural challenges and legislative misalignment amongst others, it seems that stakeholders in the book sector have realized that the benefits of AI outweigh its challenges. Findings in this study have demonstrated that publishers in Africa are aware of the benefits of AI in the production and distribution of information products, and some have started employing AI in their operations. While there are fewer cases of dedicated AI tools and machine learning systems, it is apparent that AI-enhanced tools such as Grammarly are more popular among publishers and other stakeholders in the book sector (Al Sawi and Alaa, 2024). As publishers have noted the benefits of AI tools, there is a possibility of widespread adoption of AI tools as Rogers (2003) explains that the observability of the effects of a technology influences its adoption. As AI transforms operations, drives sales and optimizes costs for innovators and early adopters, other publishers are likely to adopt the technology leading to its widespread use in Africa.

## Conclusion

This expose demonstrated that AI significantly transforms the book publishing landscape and is indeed a double-edged sword, i.e., it has both positive and negative consequences but the benefits outweigh the drawbacks. Though the book sector in Europe and Asia has made strides in integrating AI into their day-to-day operations along the value chain, Africa, unfortunately, is a slow adopter of emerging and disruptive technologies. East and West Africa are leading in the integration of artificial intelligence in their business processes whilst other countries on the continent have not done so, possibly viewing it as a threat to their existence and survival. It is important to be cognizant of the formidable challenges facing the African publishing industry most of which are beyond their control. The lack of AI policy frameworks governing the use of AI, poor infrastructure and underperforming economies hinder progress in this endeavor. Fears have been expressed over the potential loss of creativity in authors, illustrators and designers, loss of jobs and so forth but this should not sway the thinking of stakeholders. Machines can never replace the human element required in creative writing, editing, design, marketing, promotion and distribution; they do not have emotions, ethics and moral sense that facilitate the achievement of desired effects in a piece of writing. The human brain is so complicated that it cannot be replaced by AI; the technology can only complement human efforts. Publishers in Africa should leverage the affordances of AI to increase efficiency and effectiveness as it reduces the time and cost of production. It would be beneficial to educational publishers if they adopted AI to quicken the provision and availability of teaching and learning resources to the education sector. The disruptive nature of AI is likely to lead to the development of entirely new businesses that are different from the publisher's traditional mandate, and also redesign their relationships with other members of the value chain. Publishers in Africa therefore should invest in low-cost AI tools to help optimize production processes and enjoy the rewards of a digital-first approach.

## Recommendations

In light of the discussion demonstrating the transformative changes brought about by AI to the publishing industry across the globe, the following recommendations are proffered to the African publishing sector:

1. Professionals in the industry should acquire new skills to keep pace with the evolving jobs and roles constantly being redefined by emerging technologies.
2. Stakeholders should actively participate in formulating policies governing the usage of AI and ensure that they uphold ethical standards and integrity of publications.

## Future research directions

The concept of artificial intelligence and its integration into the publishing industry's value chain is quite topical and there is very little visible literature on its adoption in Africa. It would be quite beneficial if empirical studies were carried out in Southern and North Africa to gain an in-depth understanding of the publishers of AI.
